# Graphitic Carbon Nitride (C_3_N_4_) Reduces Cadmium and Arsenic Phytotoxicity and Accumulation in Rice (*Oryza sativa* L.)

**DOI:** 10.3390/nano11040839

**Published:** 2021-03-25

**Authors:** Chuanxin Ma, Yi Hao, Jian Zhao, Nubia Zuverza-Mena, Ahmed G. Meselhy, Om Parkash Dhankher, Yukui Rui, Jason C. White, Baoshan Xing

**Affiliations:** 1 Key Laboratory for City Cluster Environmental Safety and Green Development of the Ministry of Education, Institute of Environmental and Ecological Engineering, Guangdong University of Technology, Guangzhou 510006, China; chuanxin.ma@gdut.edu.cn (C.M.); hy0305hy@163.com (Y.H.); 2 The Connecticut Agricultural Experiment Station, New Haven, CT 06504, USA; Nubia.Zuverza@ct.gov; 3 Stockbridge School of Agriculture, University of Massachusetts Amherst, Amherst, MA 01003, USA; ameselhy@umass.edu (A.G.M.); Parkash@umass.edu (O.P.D.); bx@umass.edu (B.X.); 4 Ministry of Education Key Laboratory of Marine Environment and Ecology, Institute of Coastal Environmental Pollution Control, and Institute for Advanced Ocean Study, Ocean University of China, Qingdao 266100, China; zhaojian047@126.com; 5 Beijing Key Laboratory of Farmland Soil Pollution Prevention and Remediation, College of Resources and Environmental Sciences, China Agricultural University, Beijing 100193, China; ruiyukui@163.com

**Keywords:** rice, g-C_3_N_4_, synthesis, cadmium, arsenic, accumulation, metal transporters

## Abstract

The present study investigated the role of graphitic carbon nitride (C_3_N_4_) in alleviating cadmium (Cd)- and arsenic (As)-induced phytotoxicity to rice (*Oryza sativa* L.). A high-temperature pyrolysis was used to synthesize the C_3_N_4_, which was characterized by transmission electron microscopy, Fourier-transform infrared spectroscopy, and dynamic light scattering. Rice seedlings were exposed to C_3_N_4_ at 50 and 250 mg/L in half-strength Hoagland’s solution amended with or without 10 mg/L Cd or As for 14 days. Both Cd and As alone resulted in 26–38% and 49–56% decreases in rice root and shoot biomass, respectively. Exposure to 250 mg/L C_3_N_4_ alone increased the root and shoot fresh biomass by 17.5% and 25.9%, respectively. Upon coexposure, Cd + C_3_N_4_ and As + C_3_N_4_ alleviated the heavy metal-induced phytotoxicity and increased the fresh weight by 26–38% and 49–56%, respectively. Further, the addition of C_3_N_4_ decreased Cd and As accumulation in the roots by 32% and 25%, respectively, whereas the metal contents in the shoots were 30% lower in the presence of C_3_N_4_. Both As and Cd also significantly altered the macronutrient (K, P, Ca, S, and Mg) and micronutrient (Cu, Fe, Zn, and Mn) contents in rice, but these alterations were not evident in plants coexposed to C_3_N_4_. Random amplified polymorphic DNA analysis suggests that Cd significantly altered the genomic DNA of rice roots, while no difference was found in shoots. The presence of C_3_N_4_ controlled Cd and As uptake in rice by regulating transport-related genes. For example, the relative expression of the Cd transporter *OsIRT1* in roots was upregulated by approximately threefold with metal exposure, but C_3_N_4_ coamendment lowered the expression. Similar results were evident in the expression of the As transporter *OsNIP1;1* in roots. Overall, these findings facilitate the understanding of the underlying mechanisms by which carbon-based nanomaterials alleviate contaminant-induced phyto- and genotoxicity and may provide a new strategy for the reduction of heavy metal contamination in agriculture.

## 1. Introduction

Heavy metal contamination in soils has become a major threat to global agriculture due to both direct toxicity to crops and the subsequent impacts on human health [1,2,3]. Heavy metals in agricultural soils can be derived from both geogenic (soils) and anthropogenic (mining, smelting, solid waste, etc.) sources [4,5,6]. Cadmium (Cd) and arsenic (As) are two heavy metals that are commonly found in soils [7]. Due to its elemental properties, Cd can replace Ca and cause chronic Cd poisoning, called itai-itai disease [8]. Inorganic As has been classified as a human carcinogen by the United States Environmental Protection Agency (USEPA) [9] and can also cause a series of human diseases [10]. In addition, heavy metals have caused ecotoxicological effects on soil organisms (microorganisms, plants, and animals) due to their toxicity, bioaccumulation, and persistence in environments [11,12,13,14]. According to Tóth et al., the average topsoil concentrations of Cd and As in the European Union were 0.09 ± 0.11 and 3.72 ± 2.92 mg/kg, respectively [15]. Similarly, Cd and As concentrations in soils in the United States were 0.2–2 and 0.4–40 mg/kg [16,17]. Heavy metal-induced phytotoxicity to crops has been extensively investigated, with detailed studies addressing metal speciation and accumulation, bioavailability, physiological responses, crop yield, and quality, as well as plant defense mechanisms [18,19,20,21,22]. In addition, efforts have been made to stabilize heavy metal contaminants in agricultural soils using different types of amendments (minerals, organic matters, biofertilizers, rhizosphere microbial community), which have subsequently reduced metal accumulation in crops and risk of human exposure [23,24,25]. Thus, it is important to not only explore novel, sustainable, and efficient strategies to reduce heavy metal uptake but also reveal the underlying interaction and uptake mechanisms to maximize benefits.

Nano-enabled techniques have been widely used in agriculture for the purposes of monitoring plant health, enhancing crop yield, and suppressing abiotic and biotic stresses [26,27,28,29]. A number of recent studies have demonstrated positive impacts of both metal- and carbon-based nanomaterials on alleviating contaminant-induced abiotic stress and toxicity. For example, Ma et al. (2020) reported that zinc oxide (ZnO) nanoparticles (NPs) could significantly reduce the Cd and As accumulation in rice tissues when grown in metal co-contaminated rice paddies, including reduced grain contamination [7]. Similar findings of ZnO NPs alleviating heavy metal toxicity to *Leucaena leucocephala* seedlings were also reported [30]. Other metal-based NPs, such as TiO_2_ [31,32] and CuO [33], also exhibited positive impacts by alleviating heavy metal phytotoxicity and enhancing crop growth. With regard to carbon-based nanomaterials, most of the studies have been conducted to facilitate understanding of the interactions between nanomaterials and contaminants [34,35,36,37]. Only a small number of studies have evaluated the impacts of carbon-based nanomaterials on alleviating contaminant-induced toxicity to crops. For example, nanoscale biochar reduced Cd accumulation in rice and subsequently ameliorated Cd-induced phytotoxicity as measured by plant growth, pigment production, and lipid peroxidation [38]. In addition, Jia et al. (2020) reported that magnetic carbon nanotubes altered phenanthrene and associated metabolite accumulation in lettuce, suggesting this approach as a novel strategy for soil remediation [39]. Additional investigations exploring the potential of sustainable carbon-based nanomaterials to reduce heavy metal accumulation and phytotoxicity to crops are needed.

Graphitic carbon nitride nanosheets (C_3_N_4_) have attracted attention in recent years due to their unique structure and excellent catalytic properties. Containing only carbon and nitrogen, C_3_N_4_ can be easily synthesized using low-cost nitrogen-enriched compounds such as urea and melamine under heat condensation [40,41,42]. Xiao et al. (2019) reported the superior adsorption performance of C_3_N_4_ for heavy metal removal from wastewater; the maximum adsorption capacities of Cd, lead (Pb), and chromium (Cr) were approximately 123, 37, and 684 mg/g, respectively [43]. Similar results were demonstrated for C_3_N_4_ quantum dots (QD) removal of mercury chloride (HgCl_2_), with a binding efficiency of 24.63 mg HgCl_2_/10 mg C_3_N_4_ [44]. However, biotic and in vivo experiments investigating C_3_N_4_ potential for reducing heavy metal accumulation in crops are very limited. Hao et al. (2021) reported that C_3_N_4_ not only significantly reduced the Cd content of rice tissues but also increased the nitrogen content to offset the Cd-induced nitrogen deficiency [45]. However, a mechanistic understanding of C_3_N_4_ regulation of heavy metal transporters at the molecular level remains elusive.

Rice, a semiaquatic annual grass species, is the most important cereal crop in developing countries and the most consumed staple food all over the world [46]. In the present study, rice (*Oryza sativa* L.) seedlings were hydroponically exposed to C_3_N_4_ and Cd- or As-amended nutrient solutions under greenhouse conditions for 14 days. At harvest, physiological parameters and elemental content of rice tissues were measured across all treatments. In addition, the relative expression of Cd- and As-related transporters was analyzed as affected by C_3_N_4_ and both Cd and As. The findings provide important information on the role of C_3_N_4_ in reducing Cd and As bioavailability and subsequent phytotoxicity to crops. More importantly, the work further demonstrates the use of sustainable nano-enabled techniques as a novel strategy for soil remediation to ensure a safe food supply.

## 2. Materials and Methods

### 2.1. C3N4 Synthesis and Characterization

Graphitic carbon nitride nanosheets (C_3_N_4_) were synthesized with urea (20 g) in a vacuum tube furnace. The ramping rate was 5 °C/min, and the temperature was kept at 550 °C under nitrogen flow for 4 h [47]. The synthesized C_3_N_4_ was cooled down to ambient temperature in the vacuum tube furnace and then washed with deionized water three times. The C_3_N_4_ yield in each batch was approximately 2% (*w*/*w*). The procedures were repeated several times to prepare sufficient C_3_N_4_ for experimentation. All batches of C_3_N_4_ were mixed thoroughly and then freeze-dried in a lyophilizer (<1.5 mbar, −50 °C, FreeZone^®^ Benchtop Freeze Dryers, Model 70020, Labconco Corporation, Kansas City, MO, USA)

For imaging characterization, the synthesized C_3_N_4_ was dispersed in deionized water and a small volume diluted with methanol (1:1 *v*/*v*). One drop (2 μL) was deposited onto carbon-coated Cu grids and left to dry at room temperature. Images were taken in a Hitachi model HT7800 transmission electron microscope equipped with a lanthanum hexaboride (LaB_6_) filament in high contrast mode at an accelerating voltage (HV) of 80 kV. (Hitachi Incorporation, Tokyo, Japan)

For analysis by Fourier-transform infrared spectroscopy (FTIR, Spectrum One, PerkinElmer Inc, Waltham, MA, USA) equipped with the universal ATR sampling accessory, approximately 50 mg C_3_N_4_ was pressed into a thin layer and loaded onto the instrument’s crystal; the samples were scanned, and spectra of C_3_N_4_ in the range of 450–4000 cm^−1^ were collected [48].

In addition, 50 and 250 mg/L C_3_N_4_ suspensions were separately prepared in deionized water and half-strength Hoagland’s solution. Hydrodynamic diameter and zeta potential were measured by dynamic light scattering (90 Plus Particle Size Analyzer, Brookhaven, Upton, NY, USA) [48].

### 2.2. Hydroponic Experimental Design

Rice seeds (*Oryza sativa* L.) were sterilized with 70% (*v*/*v*) ethanol for 10 min and rinsed three times with deionized water. The sterilized seeds were germinated and grown in vermiculite for 2 weeks prior to the hydroponic experiment. Vermiculite on the root surfaces was gently removed in tap water, and then plants were transferred into a 100 mL glass jar containing half-strength Hoagland’s solution (mg/L: 57.52 ammonium phosphate, monobasic; 1.43 boric acid; 328.2 calcium nitrate; 0.04 cupric sulfate·5H_2_O; 1.68 Na_2_EDTA·2H_2_O; 1.3 ferrous sulfate·7H_2_O; 120.38 magnesium sulfate, anhydrous; 0.91 manganese chloride·4H_2_O; 0.008 molybdenum trioxide; 303.3 potassium nitrate; 0.11 zinc sulfate·7H_2_O, PhytoTechnology Laboratories Inc., Lenexa, KS, USA). After a 5-day acclimation period, rice seedlings were exposed to 50 and 250 mg/L C_3_N_4_ with or without 10 mg/L As (sodium arsenate, Na_3_AsO_4_) or Cd (cadmium chloride, CdCl_2_); additionally, As and Cd single analyte exposures were established as metal controls. Five biological replicates were established for each treatment. The plants were grown for 14 days. At harvest, all seedlings were rinsed with deionized water three times to remove the surface-attached C_3_N_4_ and As and Cd. The fresh biomasses of shoots and roots across all treatments were recorded, and all tissues were stored at −80 °C until further analysis.

### 2.3. Elemental Analysis of Rice Tissues

Shoot and root samples were freeze-dried in a lyophilizer and then ground into fine powder. Approximately 50 and 150 mg of root or shoot tissue were weighed into digestion tubes containing 3 mL concentrated HNO_3_. The mixtures were digested at 115 °C for 40 min in a heat block and then cooled to ambient temperature. To complete the digestion, 500 µL H_2_O_2_ was added to each tube for another 20 min of heating at 115 °C. The cooled digests were diluted to 25 mL with deionized water. Inductively coupled plasma optical emission spectrometry (ICP-OES; iCAP 6500, Thermo Fisher Scientific, Waltham, MA, USA) was used to determine the As, Cd, and nutrient element (macronutrients (P, S, Ca, Mg, and K) and micronutrients (Cu, Fe, Mn, and Zn)) contents in the acid digested samples [49]. Yttrium (Y) was used as an internal standard, and a sample of known concentration was measured at every 30 samples.

### 2.4. Real-Time Quantitative PCR Analysis of As and Cd Transporters in Rice

Fresh tissues of all five biological replicates in each treatment with 250 mg/L C_3_N_4_ were ground into a fine powder in liquid nitrogen. A Sigma-Aldrich Spectrum Plant Total RNA kit (Sigma-Aldrich Corp. St. Louis, MO, USA) was used to isolate total RNA from roots and shoots. The total RNA concentration and quality were determined by a Thermo Scientific NanoDrop Lite Spectrophotometer (NanoDrop 2000, Thermo Fisher Scientific, Waltham, MA, USA). One microgram of the extracted RNA was used as template to synthesize complementary DNA (cDNA) with a Verso cDNA synthesis kit. A complete list of primer sequences for As and Cd transporters is provided in Table S1 [50,51,52]. The synthesized cDNA was diluted to 50 ng/µL, and was used as the template for the following qPCR analysis. Bio-Rad SsoAdvanced Universal SYBR Green Supermix (Bio-Rad Incorporation, Hercules, CA, USA) was used to run the qPCR, and the working concentration of each primer was 10 µM. The thermal program profile for qPCR amplification was 95 °C for 30 s, 95 °C for 15 s, and 63 °C for 30 s, repeating 40 cycles, melting curve from 65 to 95 °C. The total volume of each reaction was 20 µL, and histone H3 was used as a housekeeping gene for normalization. The relative expression of each gene was calculated through the 2^−ΔΔCt^ method [53].

### 2.5. Random Amplified Polymorphic DNA (RAPD) Analysis

The total DNA of shoots and roots in the treatments with 250 mg/L C_3_N_4_ with or without the addition of As or Cd and the treatments with As or Cd alone were extracted using a Qiagen DNeasy Plant Mini Kit. Random (Qiagen Incorporation, Germantown, MD, USA) amplified polymorphic DNA (RAPD) analysis was performed using Taq DNA polymerase with a standard *Taq* buffer. The amplification profile was 92 °C for 1 min, 35 °C for 1 min, and 72 °C for 2 min, and the cycle was repeated 39 times. The RAPD primer used in this assay was OPC20 (ACT TCG CCA C) [54]. PCR products were run in 1% agarose gel, and images were taken under UV light in a gel dock.

### 2.6. Statistical Analysis

For each assay, the means of four to five replicates were calculated; error bars represent the standard error of the mean. A one-way analysis of variance (one-way ANOVA) followed by Duncan’s multiple comparison test was used to determine statistical significance at *p* < 0.05 across all treatments. For gene expression, a Student’s *t*-test was used to determine statistical difference (*p* < 0.05 or *p* < 0.01) between the control and each treatment.

## 3. Results and Discussion

### 3.1. C_3_N_4_ Characterization 

**Figure 1:**
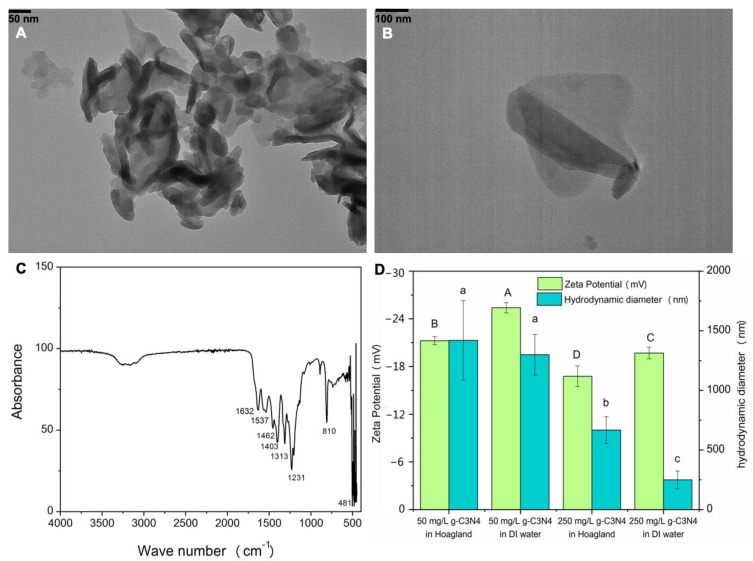
Characterization of C_3_N_4_ nanosheets. (**A**,**B**) represent TEM images of C_3_N_4_; (**C**) shows FTIR scheme 3, N_4_; (**D**) shows the hydrodynamic diameter and zeta potential of C_3_N_4_ in deionized water (DI) water and half-strength Hoagland’s solution. Values of zeta potential followed by different uppercase letters are significantly different at *p* < 0.05; values of hydrodynamic diameter followed by different lowercase letters are significantly different at *p* < 0.05.

Figure 1A,B shows the morphology of the synthesized C_3_N_4_. The FTIR spectra demonstrate characteristic peaks at 810 cm^−1^ and 1600–1200 cm^−1^ (Figure 1C), corresponding to the breathing mode of triazine units and the stretching mode of CN heterocycles, respectively [55]. All of this indicates successful C_3_N_4_ synthesis. The zeta potential value indicates that C_3_N_4_ was negatively charged (−18 to −24 mV in both deionized water and nutrient solutions); half-strength Hoagland’s solution further decreased the zeta potential as compared with deionized water. The hydrodynamic diameters of C_3_N_4_ in half-strength Hoagland’s solution and deionized water were similar at 50 mg/L. However, at 250 mg/L, the hydrodynamic diameters of C_3_N_4_ in half-strength Hoagland’s solution and deionized water were decreased to approximately 600 and 250 nm, respectively (Figure 1D). The possible explanation could be that a high concentration of C_3_N_4_ simply formed large aggregates, which could settle faster in the solution.

### 3.2. Fresh Biomass

**Figure 2:**
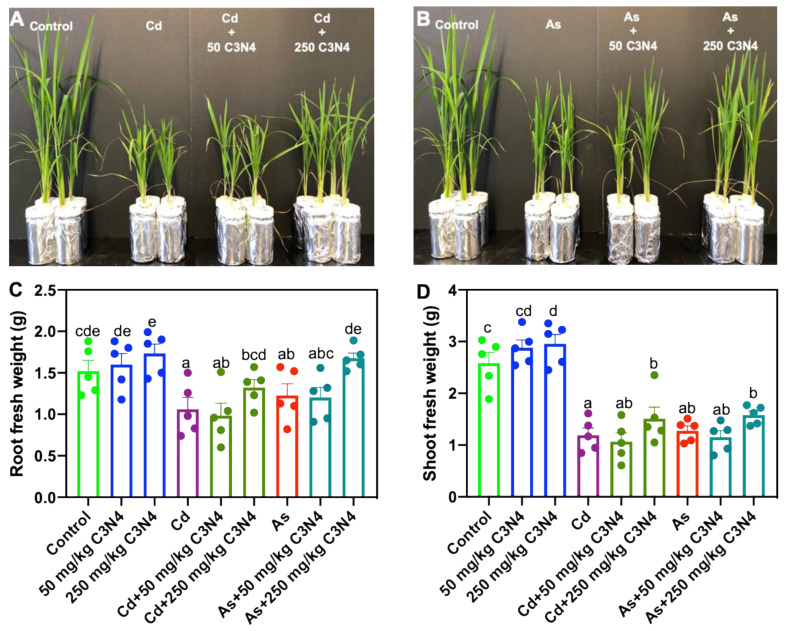
Phenotypic images and fresh weight of rice treated with Cd, As, and C_3_N_4_. (**A**,**B**) represent rice images as affected by Cd × C_3_N_4_ and As × C_3_N_4_ for 14 days, respectively. (**C**,**D**) show the fresh biomass of rice roots and shoots across all the treatments, respectively. Values of fresh weight followed by different letters are significantly different at *p* < 0.05.

After a 14-day exposure, 50 and 250 mg/L C_3_N_4_ stimulated the growth of rice as plants grew better than the untreated controls (Figure S1). In the treatments with Cd or As, phenotypic images show overt phytotoxicity to rice in terms of the shoot size; the addition of different concentrations of C_3_N_4_ alleviated both As- and Cd-induced toxicity and notably elevated the aboveground biomass (Figure 2A,B). In the C_3_N_4_ alone treatment, exposure to 250 mg/L increased the fresh mass of roots and shoots by 17.5% and 25.9%, respectively. Although the presence of 50 mg/L C_3_N_4_ also increased the fresh weight of both tissues, large variance across the five biological replicates caused the statistical significance to be elusive. In the heavy metal alone treatments, both Cd and As resulted in 26–38% and 49–56% decreases in rice root and shoot biomass, respectively, when compared with the corresponding control (Figure 2C,D). It is notable that Cd induced greater phytotoxicity to rice than As. However, the addition of different concentrations of C_3_N_4_ alleviated the heavy metal-induced toxicity, leading to significant increases in aboveground biomass in a dose-dependent fashion. The addition of 250 mg/L C_3_N_4_ increased the Cd-treated root and shoot fresh mass by approximately 47% and 50%, respectively, relative to the Cd alone treatment (Figure 2C,D). Similarly, approximately 51% and 29% increases were evident in As-treated roots and shoots upon exposure to 250 mg/L C_3_N_4_ (Figure 2C,D). Similarly, Hao et al. (2021) reported that the addition of 200 mg/L C_3_N_4_ increased the shoot height and root length of Cd-treated rice by 14% and 42%, respectively, relative to the Cd alone treatment. Additionally, the authors reported a 20% increase in rice fresh weight upon cotreatment with Cd and C_3_N_4_, but a low dose of C_3_N_4_ (20 mg/L) had no impact on enhancing the biomass of Cd-treated rice [45]. Nanoscale biochar amendment also increased the dry weight of Cd-treated rice tissues by approximately 20–40% relative to the Cd control; the pigment content in the Cd-treated rice showed a dose-dependent increase with greater nanoscale biochar doses [38]. Conversely, although alkaline fertilizer amendment with or without Mn reduced Cd accumulation in rice, the yield was not significantly altered as compared with the Cd control [56]. The coexposure of nanomaterials and an organic contaminant, TiO_2_ NPs, also alleviated the tetracycline-induced toxicity to *Arabidopsis* and rice [57,58], suggesting the significant potential of nanoscale materials as amendments for soil remediation, or alleviated heavy metal phytotoxicity.

### 3.3. Cd and As Content in Rice Tissues 

**Figure 3:**
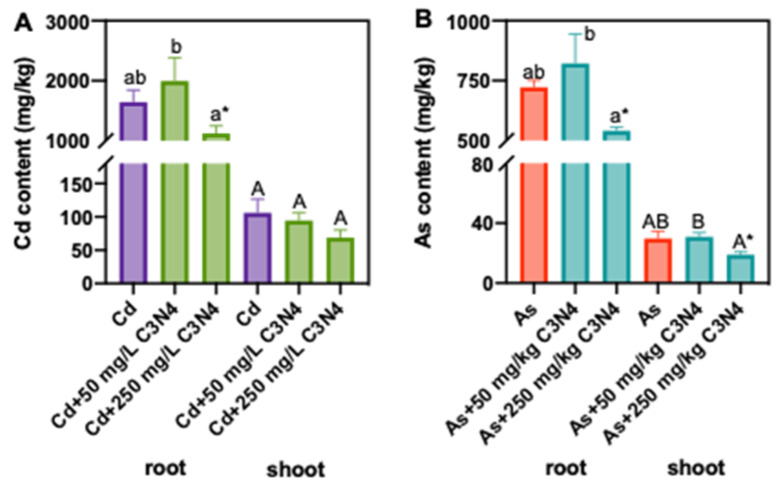
The Cd (**A**) and As (**B**) contents in rice roots and shoots upon exposure to As or Cd with or without the addition of C3N4. Values of As or Cd contents in roots followed by different lowercase letters are significantly different at *p* < 0.05; values of As or Cd contents in shoots followed by different uppercase letters are significantly different at *p* < 0.05. Single asterisk “*” indicates the significant difference between control and each treatment at *p* < 0.05 using a Student’s *t*-test.

In the As and Cd alone treatments, the shoot and root Cd contents were 105 and 1643 mg/kg, which were 3.5- and 2.3-fold of the As contents in shoot and root tissues, respectively (Figure 3). The Cd and As contents in rice tissues were reduced upon exposure to 250 mg/L C_3_N_4_, particularly the roots (Figure 3). For example, addition of 250 mg/L C_3_N_4_ decreased the Cd contents in roots and shoots by 32% and 35%, respectively, as compared with the control. However, due to large variance of the shoot Cd content, the decrease was statistically insignificant as compared with the Cd alone treatment (Figure 3A). Similarly, 250 mg/L C_3_N_4_ resulted in approximately 25% and 36% decrease in As in the roots and shoots, respectively (Figure 3B), clearly demonstrating the potential of C_3_N_4_ to alter the heavy metal and metalloid uptake and distribution in rice. It is worth noting that the Cd and As translocation factors were not significantly altered as compared with the respective metal controls (data not shown). Exposure to 50 mg/L C_3_N_4_ had no impact on Cd and As contents. Yue et al. (2020) also reported that nanoscale biochar beyond 500 mg/kg reduced the Cd content in rice tissues by more than 50% and significantly outperformed bulk-sized biochar [38]. Metal-based NPs have also been shown to decrease the bioavailability of heavy metals to crop species. Sharifan et al. (2019) demonstrated that ZnO NPs’ exposure reduced the Cd contents in lettuce roots and shoots by 49% and 30%, respectively [59]. Similarly, both bulk- and nano-sized TiO_2_ NPs reduced the Pb accumulation in rice [60]. In the current study, we hypothesize that the adsorption of As and Cd onto the C_3_N_4_ surface in the rhizosphere subsequently reduces metal bioavailability [43].

### 3.4. Macro- and Micronutrient Content Analysis in Rice Tissues

**Figure 4:**
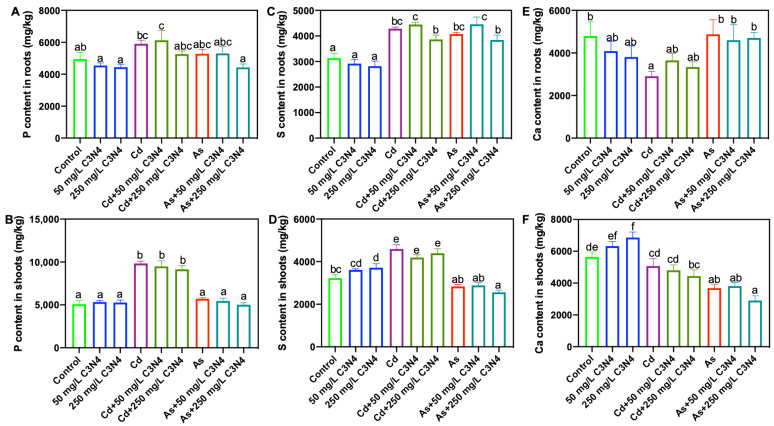
The contents of macronutrients in rice roots and shoots upon exposure to As and Cd with or without the addition of C_3_N_4_. (**A**,**C**,**E)** represent the P, S, and Ca contents in rice roots across all treatments, respectively. (**B**,**D**,**F)** represent the P, S, and Ca contents in rice shoots across all treatments, respectively. Values of each nutrient content in shoots followed by different letters are significantly different at *p* < 0.05.

Both macro- and micronutrients are involved in critical metabolism and development processes throughout the plant life cycle [61]. Upon exposure to abiotic stressors, such as heavy metals, displacement of nutrient uptake and distribution in rice could result in phytotoxicity and may trigger plant defense mechanisms. Consequently, the macro- and micronutrient contents of rice tissues upon coexposure to Cd or As and C_3_N_4_ were measured. Exposure to both Cd and As significantly altered rice root and shoot macronutrients, although the observed changes were metal specific (Figure 4 and Figure S2). For example, exposure to Cd increased shoot P content by more than 90% as compared with controls, regardless of the C_3_N_4_ presence (Figure 4B), whereas no difference was found with As treatments (Figure 4B). Similarly, the shoot S content was increased by more than 40% and 60% upon Cd treatments as compared with the control and the As treatments, respectively (Figure 4D). Exposure to As led to decreases in the tissue macronutrient content relative to the Cd treatments. For example, the lowest K contents in both roots and shoots were evident in the As-treated tissues, regardless of the presence of C_3_N_4_ (Figure S2C,D). Similar results were also observed for the shoot S, Ca, and Mg contents (Figure 4D,F and Figure S2B). Importantly, the addition of C_3_N_4_ had little impact on macronutrient content, the exception being shoot S and Ca in the 250 mg/L C_3_N_4_ treatment, where the contents were significantly higher than those in the control (Figure 4D,F).

**Figure 5:**
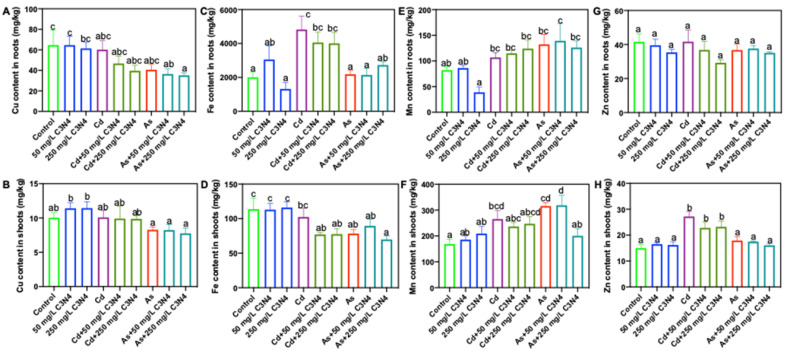
The contents of micronutrients in rice roots and shoots upon exposure to As and Cd with or without the addition of C_3_N_4_. (**A**,**C**,**E**,**G**) represent the Cu, Fe, Mn, and Zn contents in rice roots across all treatments, respectively. (**B**,**D**,**F**,**H**) represent the Cu, Fe, Mn, and Zn contents in rice shoots across all treatments, respectively. Values of each nutrient content in roots followed by different letters are significantly different at *p* < 0.05.

In general, changes to the tissue micronutrient content were of a lesser magnitude than those to the macronutrient content, although some statistically significant changes were evident (Figure 5). For example, the Cu contents in roots cotreated with As and C_3_N_4_ were significantly lower than those in the control (Figure 5A). Similarly, root Fe content in the Cd treatments with or without C_3_N_4_ was increased by more than 100% relative to the control (Figure 5C); additionally, 50–80% increases in the shoot Zn contents were found in the Cd treatments, regardless of C_3_N_4_ exposure (Figure 5G). In comparison with Cd, As exerted less impact on micronutrient displacement in rice, the exception being shoot Fe, where the Fe content was decreased by 20% relative to the control (Figure 5D). Overall, the nutrient analysis demonstrates that heavy metal exposure can induce nutrient displacement in rice and that C_3_N_4_ amendment has little impact on these alterations.

Nutrient displacement and dysfunction induced by exposure to heavy metals or other contaminants can severely compromise crop health. For example, tetracycline reduced the macronutrient (K, P, and S) contents of rice by approximately 20% relative to the control [58]. High doses of metal-based NPs, such as Ag, CeO_2_, and In_2_O_3_, can significantly decrease Fe content and subsequently compromise plant metabolism [49,62]. No published studies have reported on the levels of mineral nutrients in crops affected by C_3_N_4_ exposure. It is worth noting that the addition of C_3_N_4_ had little impact on nutrient accumulation in rice, suggesting that the mode of action may be through stabilization of heavy metals in soils.

### 3.5. Plant Molecular Response 

#### 3.5.1. RAPD Analysis upon Exposure to C_3_N_4_ and Heavy Metals

RAPD was employed to assess the potential of both C_3_N_4_ and heavy metals to induce DNA damages in rice. In shoot tissues, the amplicon sizes of all bands were between 500–1500 bp, and there were no significant changes in the total number of DNA bands across all C_3_N_4_ and heavy metal treatments relative to the control. In root tissues, the presence of Cd significantly altered the total number of DNA bands. Two additional DNA bands (>1500 bp) were evident in the Cd alone treatment, and one band (>1500 bp) was found in the cotreatment with Cd and C_3_N_4_. Conversely, the total number of DNA bands was unchanged upon As treatment (Figure S3), indicating that Cd had more negative impact on rice DNA than As. C_3_N_4_ had no significant impact on the total number of DNA bands in rice tissues. These findings align with those of Venkatachalam et al., who reported that 50 mg/L Cd caused an additional band at 1100 bp in exposed *Leucaena leucocephala* seedlings; conversely, ZnO NPs alone had no impact relative to the control [30]. Mosa et al. (2018) used three different primers (OPA7, OPA8, and OPA9) in cucumber to demonstrate that copper NPs induced additional bands as compared with the corresponding control, suggesting that copper NPs can also cause genomic alteration [63].

#### 3.5.2. Relative Expression of Cd and As Transporters 

**Figure 6:**
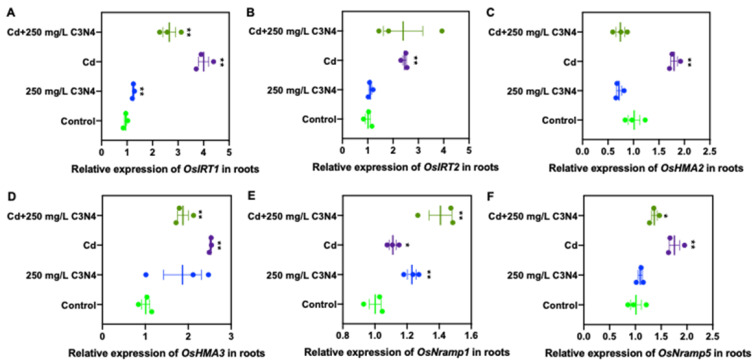
The relative expression of Cd transport-associated genes in rice roots upon exposure to Cd with or without the addition of C_3_N_4_. (**A**,**B**) represent the relative expression of Fe-regulated transporters *IRT1* and *IRT2*, respectively, in roots. (**C**,**D**) represent the relative expression of heavy metal ATPases *HMA2* and *HMA3*, which mediate the Cd loading and translocation from roots to shoots, in roots. (**E**,**F**) show the relative expression of the natural resistance-associated macrophage proteins *Nramp1* and *Nramp5*, respectively, in rice roots affected by As and C_3_N_4_. Single asterisk “*” indicates the significant difference between control and each treatment at *p* < 0.05; double asterisks “**” indicate the significant difference between control and each treatment at *p* < 0.01 using a Student’s *t*-test.

**Figure 7:**
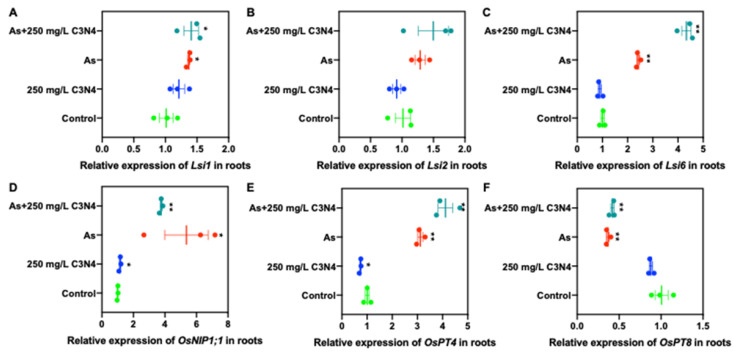
The relative expression of As transport-associated genes in rice roots upon exposure to As with or without the addition of C_3_N_4_. (**A**–**C**) represent the relative expression of the Si transport-related genes (*Lsi1*, *2*, and *6*), which have a demonstrated association with arsenite transport in roots. (**D**) shows the relative expression of nodulin 26-like intrinsic proteins (*NIPs1;1*) associated with arsenite uptake in roots. (**E**,**F)** show the relative expression of the Pht1 family genes, *OsPT1* and *OsPT8*, involving arsenate uptake, respectively, in rice roots as affected by As and C3N4. Single asterisk “*” indicates the significant difference between control and each treatment at *p* < 0.05; double asterisks “**” indicate the significant difference between control and each treatment at *p* < 0.01 using a Student’s *t*-test.

In order to explore the underlying mechanisms by which C_3_N_4_ altered heavy metal accumulation in rice, the relative expressions of Cd- and As-related transporters in root and shoot tissues were evaluated (Figure 6, Figure 7, Figure S4 and S5). The relative expression of rice iron-regulated transporter 1, *OsIRT1,* in roots in the Cd alone treatment was upregulated by approximately threefold of the control; however, the addition of C_3_N_4_ reduced this expression by 25% (Figure 6A). No difference was noted in the expression of the other *IRT* gene (*OsIRT2*) in the roots with Cd alone or with coexposure to C_3_N_4_ (Figure 6B). However, the addition of C_3_N_4_ significantly reduced the Cd-related transporter expression of rice heavy metal P-type ATPases (*OsHMA2* and *OsHMA3*) and natural resistance-associated macrophage protein 5 (*OsNramp5*) in metal-treated roots (Figure 6C,D,F). The expression of *OsNramp1* was rather insensitive to Cd exposure, with an expression increase less than 50% of the control. Although C_3_N_4_ slightly increased the *OsNramp1* expression, the increase was also less than 50% as compared with the control and the Cd alone treatment (Figure 6E). In the shoots, the relative expression of Cd-related transporters was not significantly upregulated upon exposure relative to the control (Figure S4A–E), with the exception being *OsNramp5*, whose level was approximately 50% higher than that of the control (Figure S4F).

With regard to As-related transporters (both arsenite and arsenate), exposure to As induced upregulation of rice nodulin 26-like intrinsic proteins, *OsNIP1;1*, and phosphate transporter, *OsPT4*, in the roots relative to the control (Figure 7D,E). Either the expression of the remaining genes was increased by less than 50% of the control, or no change was evident upon exposure to As and C3N4 (Figure 7A–C). Downregulation of the *OsPT8* expression was evident in both the As alone and cotreatment with C_3_N_4_ (Figure 7F). Similarly, in shoots the regulation of As-related transporters in the As treatments was similar to the control (<50% change) (Figure S5). However, the addition of C_3_N_4_ downregulated the expression of low silica transporters, *Lsi1* and *Lsi2*, and *OsPT4* in shoot tissues (Figure S5A,B,E).

The expression and function of genes involved in Cd and As transport in rice have been extensively studied. In the present work, exposure to Cd upregulated both *OsIRTs* in rice, which is consistent with the amounts of Fe and Cd detected in rice tissues. Similar results were reported by Jiang et al. (2020), who demonstrated that Cd transporter-related genes were elevated in rice upon exposure to Cd, while the presence of glutamate lowered their expression and consequently reduced the Cd uptake [52]. Ma et al. (2016) also demonstrated that the expression of Fe-related transporters in *Arabidopsis* was downregulated upon CeO_2_ NP treatment, which could explain the reduced Fe content as compared with the control [49]. Regarding As, aquaporin-related genes in wheat and tomato were notably upregulated upon exposure to graphene and As; additionally, coexposure to these two analytes could result in relatively higher expression of these genes relative to the single analyte treatments [64].

## 4. Conclusions

In summary, C_3_N_4_ significantly alleviated Cd- and As-induced phytotoxicity to rice without exerting any additional or unique negative impact on plant growth as determined by phenotype and biomass. In addition, C_3_N_4_ modulated the expression of Cd and As transporter genes and subsequently reduced contaminant accumulation or bioavailability, offering one of the mechanistic insights into the observed effects. Further investigation evaluating grain yield and quality in rice coexposed to heavy metals and C_3_N_4_ is warranted. Overall, the present work demonstrates that C_3_N_4_ nanosheets are able to alleviate the phytotoxicity and reduce the accumulation of Cd and As in rice. Therefore, the use of C_3_N_4_ is a promising material to be studied as a sustainable and safe nano-enabled strategy for reducing heavy metal accumulation in important food crops grown in contaminated soils.

## Data Availability

Data is contained within the article or supplementary material.

## Supplementary Materials

The following are available online at https://www.mdpi.com/2079-4991/11/4/839/s1, Figure S1: Phenotypic image of rice treated with 50 and 250 mg/L C3N4 for 14 days; Figure S2: The content of macronutrients (Mg and K) in rice roots and shoots upon exposure to As, Cd with or without the addition of C3N4; Figure S3: Random Amplified Polymorphic DNA (RAPD) analysis with random oligonucleotide primer OPC20; Figure S4: The relative expression of Cd transport-associated genes in rice shoots upon exposure to Cd with or without the addition of C3N4; Figure S5: The relative expression of As transport-associated genes in rice shoots upon exposure to As with or without the addition of C3N4; Table S1: A list of used primers.
