# Supplementary material for: Graphitic Carbon Nitride (C3N4) Reduces Cadmium and Arsenic Phytotoxicity and Accumulation in Rice (Oryza sativa L.)
Source: Nanomaterials (Basel). 2021 Mar 25;11(4):839. doi: 10.3390/nano11040839 (PMC8064487; doi:10.3390/nano11040839)
Supplement: Supplementary file 1 [file nanomaterials-11-00839-s001.pdf]

nanomaterials

Supporting Information

Graphitic Carbon Nitride (C_3N_4) Reduces Cadmium and Arsenic Phytotoxicity and Accumulation in rice (*Oryza sativa* L.)

Chuanxin Ma [1,2], Yi Hao [1,3], Jian Zhao [4], Nubia Zuverza-Mena [2], Ahmed G. Meselhy [3], Om Parkash Dhankher [3], Yukui Rui [5], Jason C. White [2,*] and Baoshan Xing [3]

1 Key Laboratory for City Cluster Environmental Safety and Green Development of the Ministry of Education, Institute of Environmental and Ecological Engineering, Guangdong University of Technology, Guangzhou 510006, China; chuanxin.ma@gdut.edu.cn (C.M.); hy0305hy@163.com (Y.H.)
2 The Connecticut Agricultural Experiment Station, New Haven, CT 06504, USA; Nubia.Zuverza@ct.gov
3 Stockbridge School of Agriculture, University of Massachusetts Amherst, Amherst, MA 01003, USA; ameselhy@umass.edu (A.G.M.); Parkash@umass.edu (O.P.D.); bx@umass.edu (B.X.)
4 Institute of Coastal Environmental Pollution Control, Ministry of Education Key Laboratory of Marine Environment and Ecology, and Institute for Advanced Ocean Study, Ocean University of China, Qingdao 266100, China; zhaojian047@126.com
5 Beijing Key Laboratory of Farmland Soil Pollution Prevention and Remediation, College of Resources and Environmental Sciences, China Agricultural University, Beijing 100193, China; ruiyukui@163.com
* Correspondence: jason.white@ct.gov

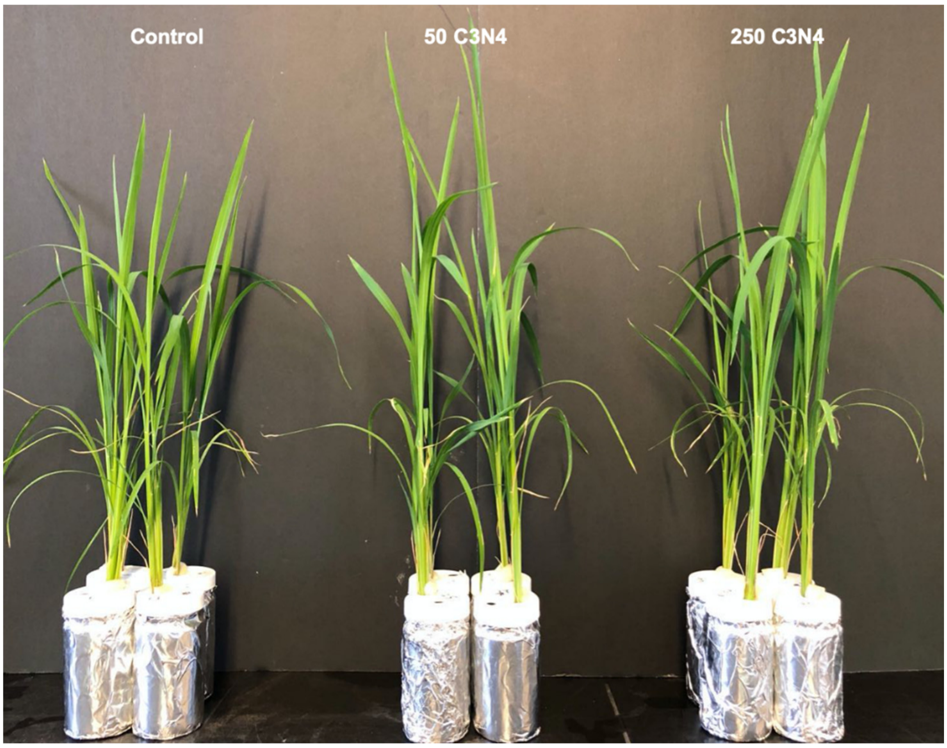

Figure S1. Phenotypic image of rice treated with 50 and 250 mg/L C_3N_4 for 14 days.

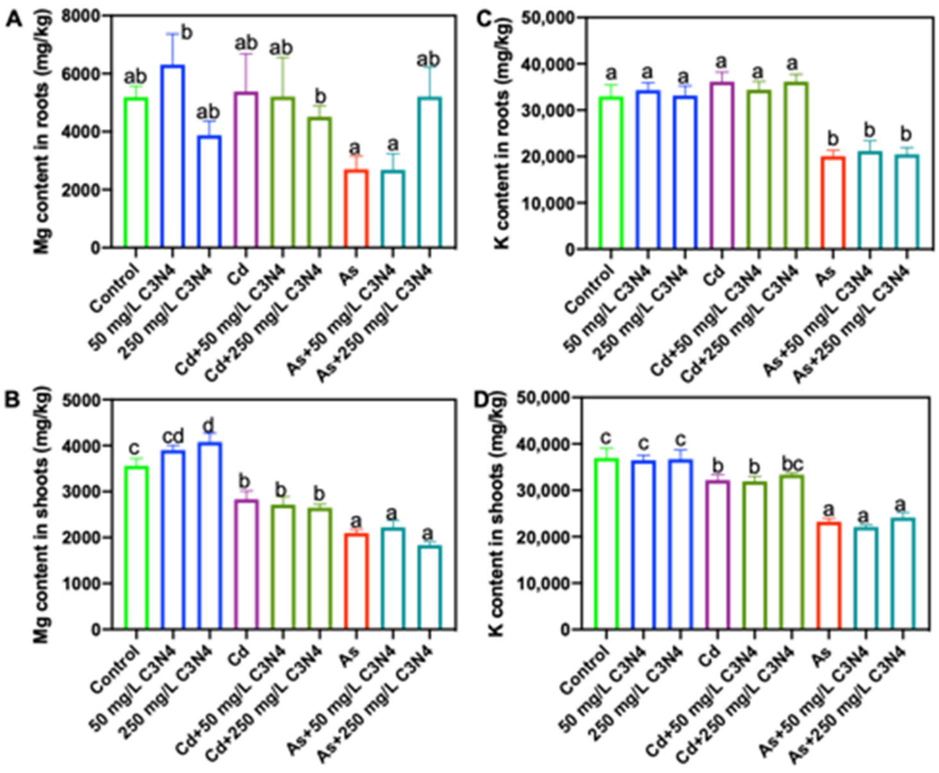

Figure S2. The content of macronutrients (Mg and K) in rice roots and shoots upon exposure to As, Cd with or without the addition of C_3N_4. (**A**,**B**) represent the Mg content in rice roots and shoots across all treatments, respectively. (**C**,**D**) represent the K content in rice shoots across all treatments, respectively. Values of each nutrient content in rice tissues followed by different letters are significantly different at $p < 0.05$.

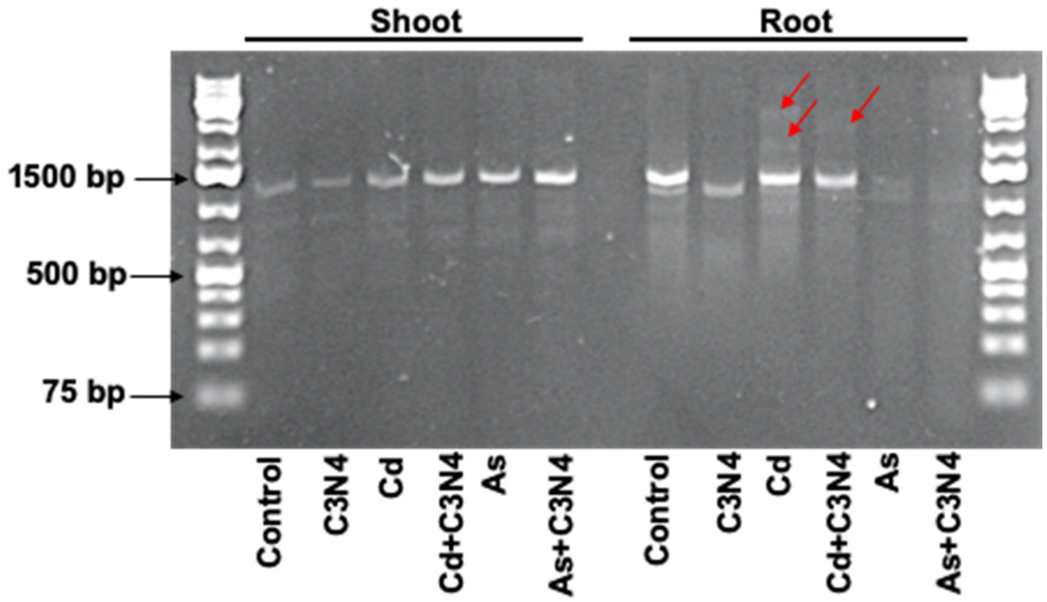

Figure S3. Random Amplified Polymorphic DNA (RAPD) analysis with random oligonucleotide primer OPC20. Additional DNA bands are shown with arrows.

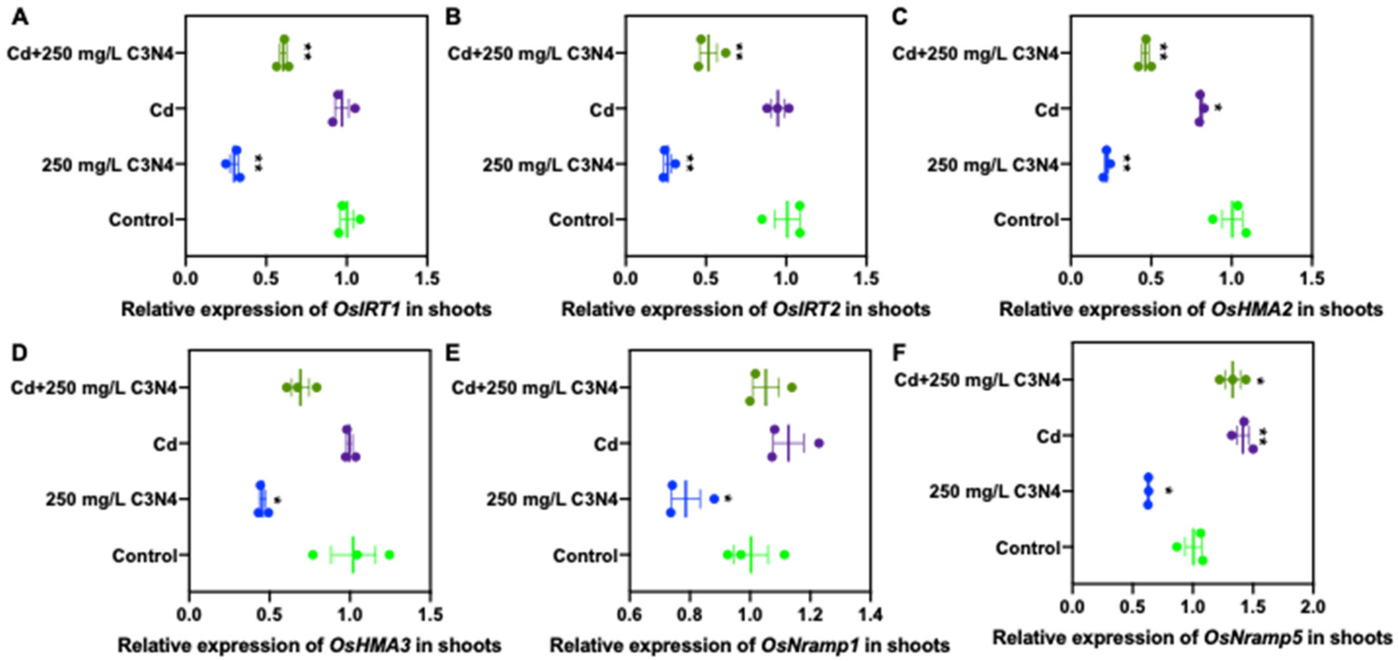

Figure S4. The relative expression of Cd transport-associated genes in rice shoots upon exposure to Cd with or without the addition of C_3N_4. (**A**,**B**) represent the relative expression of Fe-regulated transporter (*IRT1* and *IRT2*), respectively, in shoots. (**C**,**D**) represent the relative expression of heavy metal ATPase (*HMA2* and *HMA3*), which mediates the Cd loading and translocation from roots to shoots, in shoots. **(E**,**F)** show the relative expression of the natural resistance-associated macrophage protein (*Nramp1* and *Nramp5*), respectively, in rice shoots as affected by As and C_3N_4. Single asterisk '*' indicates the significant difference between control and each treatment at p <0.05; double asterisks '**' indicate the significant difference between control and each treatment at p <0.01 using a Student t-test.

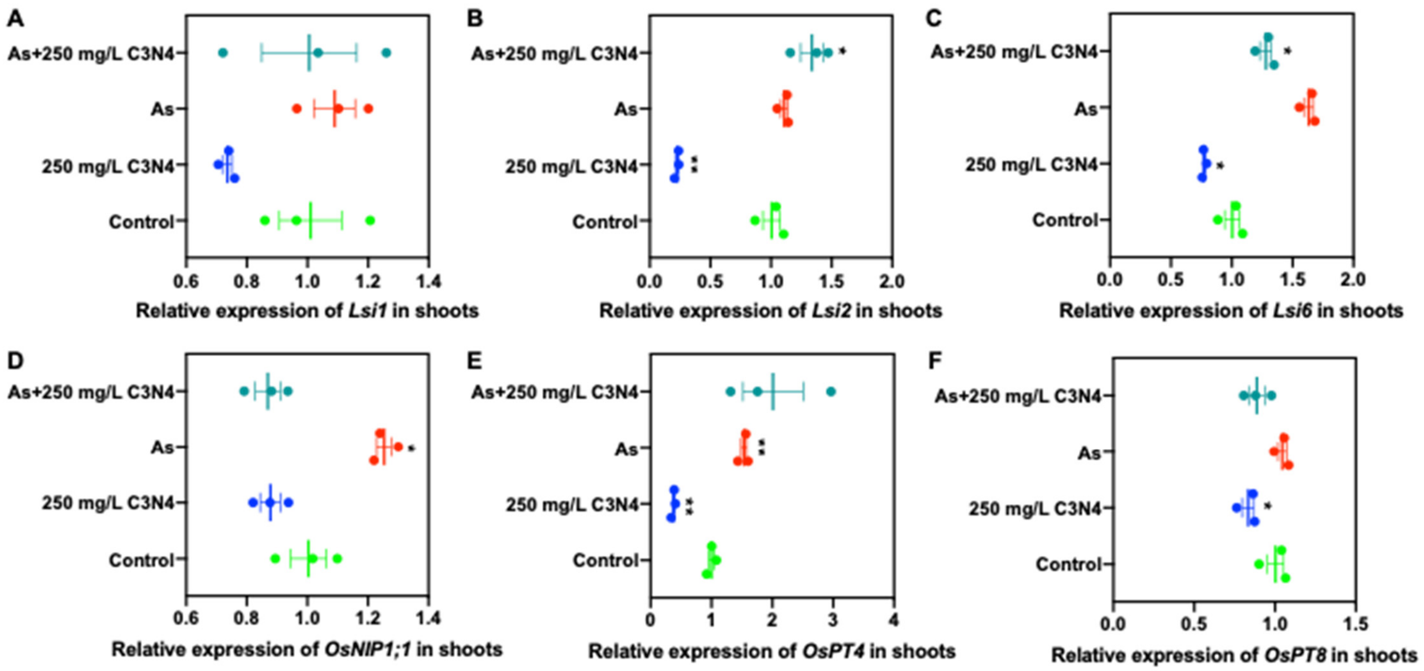

Figure S5. The relative expression of As transport-associated genes in rice shoots upon exposure to As with or without the addition of C_3N_4. (**A–C)** represent the relative expression of the Si transport related genes (*Lsi1*, *2* and *6*), which have a demonstrated association with arsenite transport in shoots. (**D)** shows the relative expression of nodulin 26-like intrinsic membrane proteins (*NIPs*1;1) associate with arsenite uptake in shoots. (**E**,**F)** show the relative expression of the Pht1 family genes, *OsPT1* and *OsPT8*, involving arsenate uptake, respectively, in rice shoots as affected by As and C_3N_4. Single asterisk '*' indicates the significant difference between control and each treatment at $p < 0.05$; double asterisks '**' indicate the significant difference between control and each treatment at $p < 0.01$ using a Student t-test.

Table S1. A list of used primers.

Sequence Name	Sequence	Assay
Lsi1 F	CGG TGG ATG TGA TCG GAA CCA	
Lsi1 R	CGT CGA ACT TGT TGC TCG CCA	
Lsi6 F	GAG TTC GAC AAC GTC TAA TCG C	
Lsi6 R	AGT ACA CGG TAC ATG TAT ACA CG	
OsNIP1;1 F	CTG ATT GCT GGG CCG ATC TCG	
OsNIP1;1 R	GCA GTA GTA GTA CTG GCA GTA G	
Lsi2 F	ATC TGG GAC TTC ATG GCC C	
Lsi2 R	ACG TTT GAT GCG AGG TTG G	
HistoneH3 F	AGT TTG GTC GCT CTC GAT TTC G	
HistoneH3 R	TCA ACA AGT TGA CCA CGT CAC G	
OsPT4 F	GCA ACG TCA TCG GGT TCT TCT TCA	
OsPT4 R	ACA TCG TCA TCG TCC TCG TTC TCG	
OsPT8 F	TCC AGA AGG ACA TCT TCA CCA GCA	
OsPT8 R	ATG TCG ATG AGG AAG ACG GTG AAC	qPCR
OsNramp1 F	CAT CGC ATA CCT TGA TCC TAG T	
OsNramp1 R	GGA GTA CCC ATA GCA ACG AAT A	
OsNramp5 F	TTC GTT TAT ATT TGT GCG GTC C	
OsNramp5 R	CAC CTC CCC TCA AAT GCT TAT A	
OsIRT1 F	GCA ATT CGC TGC ATT GTT AGA T	
OsIRT1 R	GAG AAG TCA CAG TCA CTG TAC A	
OsIRT2 F	CTT CCA CCA GAT GTT CGA GG	
OsIRT2 R	GGT GGA GAA GAA GAA GAC CAG	
OsHMA2 F	ATA CTC ATG CTG ATT GCT GGT A	
OsHMA2 R	CAA GCC AAA ATG CAT GCA TTA G	
OsHMA3 F	CAA TGG TGT TGG TCG TTG C	
OsHMA3 R	CTC CCA TTT CTG CAG TCT TTC	
OsLCT1 F	AGC ACA TCT CTG GCT TCC AC	
OsLCT1 R	CGG CTC ATT GCA TTC TGC TC	
OPC20	ACT TCG CCA C	RAPD
